# Performance of interferon-gamma levels may lead to earlier diagnosing macrophage activation syndrome complicating systemic juvenile idiopathic arthritis

**DOI:** 10.1186/s12969-023-00907-7

**Published:** 2023-10-12

**Authors:** Meiping Lu, Liping Teng, Yiping Xu, Xuefeng Xu

**Affiliations:** Department of Rheumatology Immunology & Allergy, Children’s Hospital, Zhejiang University School of Medicine, National Clinical Research Center for Child Health, Binsheng Rd 3333, Binjiang District, Hangzhou, 310052 PR China

**Keywords:** Autoinflammatory Disease, Cytokine, Diagnosis, Macrophage activation syndrome, Systemic juvenile idiopathic arthritis

## Abstract

Macrophage activation syndrome (MAS) is a severe, potentially fatal complication of rheumatic diseases, predominantly in systemic juvenile idiopathic arthritis (SJIA), and is considered as an autoinflammatory disease. Specific cytokine profiles could play a pivotal role in this inflammatory response. Gram-negative bacteremia, bacterial pneumonia, Kawasaki disease, and active SJIA exhibited similar cytokine profiles with elevated interleukin-6 (IL-6) and/or IL-10, further suggesting a correlation between them. Only when JIA is complicated by MAS can increased interferon-γ (IFN-γ) levels be observed. Therefore, increased serum IFN-γ levels could contribute to early diagnosing MAS in patients with SJIA in combination with other variables such as serum ferritin. A prospective multi-center study will be performed to further confirm the role of IFN-γ in the early recognition of MAS in SJIA.

## Main text

Macrophage activation syndrome (MAS) is a severe, potentially fatal complication of rheumatic diseases, predominantly in systemic juvenile idiopathic arthritis (SJIA). SJIA is a specific disease entity and has been recently considered as an autoinflammatory disease (AID) other than classic autoimmune diseases [1]. The majority of AIDs present with fever, rashes, and elevated acute phase proteins, including increased leukocyte and neutrophil counts, raised C reactive protein and erythrocyte sedimentation rates, which appears to be consistent with clinical manifestations of bacterial infections. Given the association of clinical features between AIDs and infections, we hypothesize that these similar clinical manifestations may be involved in certain common inflammatory or immune responses. Furthermore, the specific cytokine profile could play a pivotal role in this inflammatory response. Here, we first explored the role of cytokines in inflammation from the perspective of infection.

For children with malignancy, severe infections are a major threat to survival, and early and timely identification is crucial for prognosis. A study by Xu et al. demonstrated that IL-6 and IL-10 showed better performance in identifying gram-negative bacteremia in pediatric cancer patients, and the elevation of IL-6 and IL-10 was strongly associated with the development of gram-negative bacteremia and septic shock (Table 1) [2]. Similarly, among patients without immunodeficiency disorders who developed community-acquired pneumonia, significantly elevated serum IL-6 levels and mildly increased IL-10 levels were observed in bacterial pneumonia (Table 1). Notably, serum IFN-γ levels from both groups of cases were within the normal ranges (Table 1), regardless of their immune status. However, our study revealed that moderately elevated IL-6, IL-10, and IFN-γ demonstrated a higher prediction of the development of *M. pneumoniae* pneumonia, suggesting that a stronger immune response could be involved in the pathogenic mechanism of *M. pneumoniae* infection (Table 1) [3]. The phenomenon of increased IL-6, IL-10 and IFN-γ levels in *M. pneumoniae* pneumonia further confirmed the fact that *M. pneumoniae* infections have a dual pathogenic mechanism, including direct effects of infection and immune reactions.

**Table 1:** Comparison of serum IL-6, IL-10 and IFN-γ levels in different diseases

		Experimental group			Control group		
Authors	Disorders	IL-6 (1.7–16.6 pg/ml)	IL-10 (2.6–4.9 pg/ml)	IFN-γ (1.6–17.3 pg/ml)	IL-6 (1.7–16.6 pg/ml)	IL-10 (2.6–4.9 pg/ml)	IFN-γ (1.6–17.3 pg/ml)
Xu et al.^2^	Gram-negative bacteremia vs. non-bloodstream infections	179.3	22.3	8.3	52.9	6.3	7.7
	Bacterial pneumonia vs. viral pneumonia	884. 6	11.6	10.0	49.1	8.7	13.2
Xu et al.^3^	*M. pneumoniae* pneumonia vs. viral pneumonia	334.3	8.0	29.1	49.1	8.7	13.2
Li et al.^4^	KD shock syndrome vs. KD	184.1	42.6	18.3	54.1	9.4	6.7
Guo et al.^5^	MAS vs. active SJIA	53.4	26.9	83.1	47.2	4.2	5.3
Xu et al.^6^	HLH vs. healthy control	51.1	623.5	1088.5	5.5	6.7	5.2

KD, Kawasaki disease; HLH, hemophagocytic lymphohistiocytosis; MAS, macrophage activation syndrome; SJIA, systemic juvenile idiopathic arthritis

Kawasaki disease (KD) is a self-limited vasculitis of unknown etiology affecting children younger than 5 years of age. The overlap of clinical presentations between KD and AIDs has suggested KD as AID. Our previous study indicated that children with KD had elevated IL-6 and IL-10 levels with normal IFN-γ levels (Table 1), similar to the cytokine profile of bacterial infections. When KD patients were complicated by KD shock syndrome, they exhibited elevated IL-6, IL-10 and IFN-γ levels, further indicating the involvement of immune responses [4].

SJIA may occur at any age from childhood to adolescence. Although the seasonal distribution and non-articular systemic features of SJIA make the possibility of infection etiology attractive, there is no evidence to strengthen this hypothesis. The characteristic manifestations of SJIA including typical spiking fevers, skin rash, raised erythrocyte sedimentation rate and fibrinogen can be explained by an inflammatory response involving the cytokines IL-1 and IL-6. Our study also showed that patients with active SJIA exhibited elevated serum IL-6 levels, accompanied by normal IL-10 and IFN-γ levels. However, when those with active SJIA progressed into macrophage activation syndrome (MAS), they presented with increased serum IL-6, IL-10, and IFN-γ levels [5].

Interestingly, MAS may provide another clue to understanding the distinctive pathogenetic features in SJIA. MAS is a severe, potentially fatal complication of rheumatic diseases, and is characterized by the excessive activation of well-differentiated macrophages, resulting in fever, hepatosplenomegaly, lymphadenopathy, severe cytopenia, serious liver damage, intravascular coagulation and neurological involvement. MAS bears a close resemblance to a group of histiocytic disorders known as hemophagocytic lymphohistiocytosis (HLH), a better-defined entity seen in a heterogeneous group of diseases including infections, tumors, and autoimmune diseases. Highly elevated serum IFN-γ and IL-10 levels with moderately elevated IL-6 show high diagnostic accuracy for HLH among febrile patients and can be a valuable contribution to diagnostic criteria for HLH [6]. Although viral infections can cause fever, splenomegaly, cytopenia, and hepatocellular dysfunction, they are rarely associated with high levels of IFN-γ and IL-10 unless progressing into HLH.

Taken together, gram-negative bacteremia, bacterial pneumonia, KD, and active SJIA exhibited similar cytokine profiles with elevated IL-6 and/or IL-10, further suggesting the correlation between them. Notably, only when JIA is complicated by MAS can increased IFN-γ levels be observed. Neutralization of IFN-γ induces remission of MAS [7, 8]. Therefore, increased serum IFN-γ levels could contribute to early diagnosing MAS in patients with SJIA in combination with other variables such as serum ferritin [9]. A prospective multi-center study will be performed to further confirm the role of IFN-γ in the early recognition of MAS in SJIA.

## Data Availability

Not applicable.

## Abbreviations

AID: Autoinflammatory disease
HLH: Hemophagocytic lymphohistiocytosis
IFN: Interferon
IL: Interleukin
KD: Kawasaki disease
MAS: Macrophage activation syndrome
SJIA: Systemic juvenile idiopathic arthritis
